# Nephrologist’s Perceptions of Risk of Severe Chronic Kidney Disease and Outpatient Follow-up After Hospitalization With AKI: Multinational Randomized Survey Study

**DOI:** 10.1177/20543581251336548

**Published:** 2025-04-30

**Authors:** Dilaram Acharya, Tayler D. Scory, Nusrat Shommu, Maoliosa Donald, Tyrone G. Harrison, Jonathan S. Murray, Simon Sawhney, Edward D. Siew, Neesh Pannu, Matthew T. James

**Affiliations:** 1 Department of Medicine, Cumming School of Medicine, University of Calgary, AB, Canada; 2 Department of Community Health Sciences, Cumming School of Medicine, University of Calgary, AB, Canada; 3 O’Brien Institute for Public Health, Cumming School of Medicine, University of Calgary, AB, Canada; 4 Libin Cardiovascular Institute, Cumming School of Medicine, University of Calgary, AB, Canada; 5 South Tees Hospitals NHS Foundation Trust, Middlesbrough, UK; 6 Aberdeen Centre for Health Data Science, School of Medicine, Medical Sciences and Nutrition, University of Aberdeen, UK; 7 Division of Nephrology and Hypertension, Vanderbilt University Medical Center, Nashville, TN, USA; 8 Department of Medicine, Faculty of Medicine and Dentistry, University of Alberta, Edmonton, Canada

**Keywords:** acute kidney disease, chronic kidney disease, kidney health, nephrologist follow-up, risk stratification

## Abstract

**Background:**

Patients hospitalized with acute kidney injury (AKI) have variable risks for chronic kidney disease (CKD); however, there is limited knowledge about how this risk influences outpatient follow-up with nephrologists.

**Objective:**

This survey study examined the likelihood that nephrologists would recommend outpatient follow-up of patients with varying risk profiles for CKD after hospitalization with AKI and the effect of reporting the predicted risk of severe CKD on their decision-making.

**Design:**

A randomized survey study examining the impact of providing predicted risks of severe CKD on nephrologists’ follow-up recommendations for patients with AKI.

**Setting:**

The study included nephrologists from the United States, the United Kingdom, and Canada between September and December 2023.

**Patients:**

Participants reviewed clinical vignettes of patients with AKI and varying risks of severe CKD (G4 or G5), using an externally validated prediction model.

**Measurements:**

The primary outcome was the likelihood of recommending nephrologist specialist follow-up for each case, scored on a 7-point Likert scale (1 = “definitely not” and 7 = “definitely would”).

**Methods:**

Participants were randomized to receive a version of the survey either with or without the predicted risk of severe CKD included for each vignette. Responses were compared across categories of predicted risk (<10%, 10%-49%, and ≥50%) using generalized estimating equations.

**Results:**

Of the 203 nephrologists who participated, 73 (36%) were from the United Kingdom, 71 (35%) from Canada, and 45 (22%) from the United States. Mean (95% confidence interval [CI]) Likert scores increased from 4.01 (3.68, 4.34) for patients with a <10% predicted risk to 6.06 (5.76, 6.37) for those with a ≥ 50% predicted risk of severe CKD. Nephrologists were significantly less likely to recommend outpatient nephrology follow-up for patients with a <10% predicted risk of severe CKD when the risk was reported (mean difference = −0.71 [95% CI = −1.19, −0.23]), and significantly more likely to recommend follow-up for patients with a ≥50% predicted risk when the risk of severe CKD was reported (mean difference = 0.49 [95% CI = 0.04, 0.93]).

**Limitations:**

This study focuses on nephrologists from high-income countries and relies on hypothetical scenarios rather than real-world practices. Survey respondents may not be representative of all nephrologists, although consistent findings across diverse subgroups strengthen findings.

**Conclusions:**

When the predicted risk of severe CKD is reported, nephrologists are less likely to recommend follow-up for lower risk patients with AKI and more likely to recommend follow-up for higher risk patients, leading to better alignment of recommendations for outpatient follow-up with patient risk of severe CKD.

## Résumé

**Contexte:**

Les patients hospitalisés pour insuffisance rénale aiguë (IRA) présentent un risque variable d’évoluer vers l’insuffisance rénale chronique (IRC). On ignore toutefois si ce risque influence la décision des néphrologues de recommander un suivi en ambulatoire.

**Objectif:**

Étude par sondage examinant la probabilité que les néphrologues recommandent un suivi en ambulatoire à des patients hospitalisés pour IRA et présentant divers profils de risque d’évoluer vers l’IRC. On souhaitait également vérifier si la mention du risque prévu d’IRC grave influençait la prise de décision.

**Conception:**

Étude randomisée examinant l’impact de la divulgation du risque prévu d’IRC grave des patients hospitalisés pour IRA sur la possibilité qu’ils soient aiguillés pour un suivi en ambulatoire par un néphrologue.

**Cadre:**

Sondage auprès de néphrologues des États-Unis, du Royaume-Uni et du Canada entre septembre et décembre 2023.

**Sujets:**

À l’aide d’un modèle prédictif validé en externe, les néphrologues participants ont examiné les cas cliniques de patients hospitalisés pour IRA et présentant des risques variables d’évoluer vers l’IRC grave (G4 ou G5).

**Mesures:**

Pour chaque cas, le principal critère d’évaluation était la probabilité de recommander un suivi spécialisé par un néphrologue, évaluée à l’aide d’une échelle de Likert à sept points (1 = « certainement pas », 7 = « très certainement »).

**Méthodologie:**

Les néphrologues participants ont été répartis aléatoirement pour recevoir une version du sondage avec ou sans mention du risque prévu d’IRC grave pour chaque cas clinique. Les réponses ont été comparées selon la catégorie de risque prévu (<10 %, 10 à 49 % et ≥50 %) à l’aide d’équations d’estimation généralisées.

**Résultats:**

Des 203 néphrologues participants, 73 (36 %) provenaient du Royaume-Uni, 71 (35 %) du Canada et 45 (22 %) des États-Unis. Les scores de Likert moyens sont passés de 4,01 (IC95: 3,68 à 4,34), pour les cas où le risque prévu d’IRC grave était inférieur à 10 %, à 6,06 (IC95: 5,76 à 6,37) pour les cas où ce risque était égal ou supérieur à 50 %. Lorsque le risque prévu d’IRC grave était indiqué, les néphrologues étaient significativement moins enclins à recommander un suivi en ambulatoire aux patients présentant un risque prévu inférieur à 10 % (diff. moy.: -0,71 [IC95: -1,19 à - 0,23]) et plus enclins à le faire si ce risque était d’au moins 50 % (diff. moy.: 0,49 [IC95: 0,04 à 0,93]).

**Limites:**

L’étude repose sur des scénarios hypothétiques plutôt que sur des pratiques réelles. Seuls des néphrologues provenant de pays à revenu élevé ont été interrogés; ces derniers pourraient ne pas être représentatifs de l’ensemble des néphrologues, bien que des résultats comparables obtenus dans divers sous-groupes renforcent les résultats de la présente étude.

**Conclusion:**

Dans les cas où le risque prévu d’évolution vers l’IRC grave est indiqué, les néphrologues sont moins enclins à recommander un suivi aux patients hospitalisés pour IRA dont le risque prévu d’IRC grave est faible, et plus enclins à le faire pour les patients dont le risque prévu est élevé. Ceci permet une plus grande cohérence dans les recommandations de suivi ambulatoire pour les patients présentant un risque d’évoluer vers l’IRC grave.

## Introduction

Acute kidney injury (AKI) is a significant global public health challenge, estimated to occur in over 13 million individuals worldwide each year.^1^ AKI is prevalent among hospitalized patients, affecting approximately 18% of general inpatients and is also an independent risk factor for adverse health outcomes, including the development and progression of chronic kidney disease (CKD), cardiovascular events, kidney failure, and death.^2,3^ Recent research illustrates that almost half of patients hospitalized with AKI receive no outpatient monitoring or follow-up after discharge.^4,5^ This issue arises from many challenges in current care models, including irregular communication between hospital and community providers and under-recognition of long-term risks associated with AKI. These factors may impede proactive steps to improve the continuity of care and reduce long-term kidney and cardiovascular risk.^6-10^ Clinical practice guidelines and quality improvement recommendations suggest reassessment of kidney function after hospital discharge accompanied by clinical evaluation of volume status, blood pressure, and medication review and reconciliation to ensure that appropriate therapies for cardiovascular risk reduction and to slow CKD progression are prescribed.^11^

One strategy to improve outcomes following AKI involves risk stratification; identifying patients at increased risk of adverse long-term outcomes to optimize their care after discharge. Several routinely measured risk factors have been shown to be associated with the risk of developing severe CKD and have been combined within multivariable prognostic risk prediction models.^5,12^ However, it remains unclear how these factors influence the decisions of kidney specialists about which patients would benefit from follow-up after discharge, and whether recognition of the predicted risk of severe CKD following AKI would influence physician recommendations to provide follow-up nephrology specialty care.

To inform future care pathways intended to improve transitions of care for people after hospitalization with AKI, we performed this multinational survey study to understand how CKD risk profile influences nephrologist decision-making around the follow-up of patients after a hospitalization with AKI and to explore what effect reporting the predicted risk of severe CKD would have on their decision-making.

## Methods

### Study Design, Setting, and Survey Distribution

We conducted a randomized, survey study of nephrologists from Canada, the United Kingdom, and the United States, which was based on clinical vignettes and distributed via the Canadian Society of Nephrology, the UK Kidney Association, and the National Kidney Foundation of the United States. The survey was disseminated via a series of 3 emails sent to physician members of each organization between September and December 2023, and the analyses were completed between March and July 2024.

### Development of the Survey and Test Instruments

Two nephrologists and a researcher with survey experience designed the survey, which was refined through multiple iterations informed by pilot testing to establish content validity and clarity. Three additional nephrologists pilot tested the survey, including one from each of the countries included in the study.

The survey was administered online via Qualtrics (Qualtrics, Provo, Utah).^13^ The initial set of questions collected demographic and clinical practice information from respondents. The second section of the survey included 3 core clinical vignettes, each with 4 varying scenarios of patient characteristics, resulting in 12 unique patient scenarios that varied according to patients characteristics for age, sex, baseline estimated glomerular filtration rate (eGFR) prior to hospitalization, presence of albuminuria,^12^ Kidney Disease: Improving Global Outcomes (KDIGO) stage of AKI (based on serum creatinine [SCr]), and kidney function at the time of hospital discharge. These questions were designed to reflect a range of risk profiles across the 12 scenarios presented, with the predicted risk of severe CKD ranging from 4% to 70% based on a previously validated prediction model.^12^ Respondents were asked to indicate their likelihood of recommending outpatient follow-up with a nephrology specialist in each scenario using a 7-point modified Likert scale, ranging from 1 (definitely not) to 7 (definitely would).^14^

The last section of the survey provided 13 different patient factors that encompassed socio-demographic, clinical, and health care delivery system variables, and asked participants the degree to which each would influence their decision to arrange outpatient nephrologist follow-up for a patient after hospitalization with AKI. Responses to these questions were collected using a similar Likert scale ranging from 1 (definitely not) to 7 (definitely would) (S1: Survey questionnaires).

### Reporting of the Risk of Severe Chronic Kidney Disease

To examine the effect of reporting the predicted risk of severe (G4 or G5) CKD on physician decision-making, participants were randomly assigned at a 1:1 ratio to receive either a version of the survey with the 12 clinical scenarios accompanied by reporting of the predicted 1 year risk of severe (G4 or G5) CKD according to the risk prediction model versus an otherwise identical survey with the same clinical vignettes but no reporting of the predicted risk of severe CKD.

### Measurement of Outcome Variables and Explanatory Variables

The primary analysis focused on the effect of reporting the predicted risk of CKD on nephrologist responses, and whether this varied across the range of predicted risk of CKD presented in the scenarios. Additional descriptive analyses explored the extent to which the 13 listed socio-demographic, clinical, and health care delivery system variables influenced nephrologist responses about outpatient nephrology follow-up after hospitalization with AKI.

### Statistical Analysis

Characteristics of respondents were summarized using frequencies and percentages. Likert score responses among participants randomized to receive reporting of the predicted risk of CKD, versus those who received no reporting of the predicted risk of CKD, were graphed as a proportion of responses in each CKD risk category. A generalized linear model with an identity link was used to estimate the mean difference in Likert score responses between respondents assigned to receive the CKD risk reported versus those who were assigned to receive no CKD risk reported. Multiple responses per participant were accounted for by a random intercept and variation between CKD risk score levels were accounted for by a random slope. The final model included an interaction term between the reporting assignment group and the predicted CKD risk of each vignette, with results presented by CKD risk categories of <10%, 10% to 19%, 20% to 49%, and ≥50% predicted risk. In additional analyses, responses on the 7-point Likert scale were dichotomized as ≥5 vs <5 and a mixed-effects logistic regression model was fit to estimate the odds of a Likert score response ≥5. Mean differences and odds ratios (ORs) were presented with 95% confidence intervals (CIs), and *P* < .05 was considered statistically significant. Analyses were conducted using SAS 9.4 (SAS Institute Inc., Cary, North Carolina).

Based on the survey design for the clinical vignettes including 12 responses per participant, a sample size of 186 participants was needed to achieve 90% power to detect a mean difference of 1.0 in the response score, assuming a standard deviation between responses for each participant of 2.0, an intra-cluster correlation (ICC) of 0.50 for within respondent correlation, and with a type 1 error rate of 0.05.

### Ethical Considerations

Informed consent was obtained from all participants before completing the survey. Ethics approval for the study was obtained from the University of Calgary Conjoint Health Research Ethics Board (CHREB) (approval number: REB23-1028).

## Results

### Survey Dissemination and Respondents

**Figure 1.**
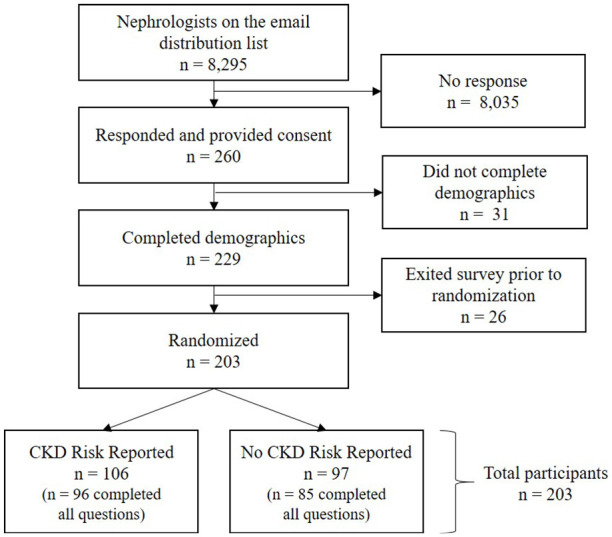
Flow diagram of nephrologist sampling, inclusion, and allocation. *Note.* CKD = chronic kidney disease.

From a collective pool of 8295 nephrologists on organization distribution lists (300 from the Canadian Society of Nephrology, 1745 from the UK Kidney Association, and 6250 from the US National Kidney Foundation), 260 (3.1%) participants responded to the invitation and provided consent to participate in the survey (Figure 1). Fifty-seven initial respondents were excluded due to exiting the survey before being randomized to one of the survey groups. From the 203 (78%) participants who were included in the study, 106 were randomized to the group who received CKD risk reporting and 97 to the group who received no CKD risk reporting and were included in the analyses.

### Demographic and Clinical Practice Characteristics of Respondents

Of the total 203 participants, 73 (36%) were from the United Kingdom, 71 (35%) from Canada, and 45 (22%) from the United States (Table 1). Nearly half reported having been in clinical practice for ≥20 years, more than 3 quarters indicated that they conducted ≥ 40 AKI consultations per year, and 78% worked in academic settings. Characteristics were similar between the groups that did and did not receive CKD risk reporting.

**Table 1.** Characteristics of Nephrologists Who Responded to the Survey.

Characteristic	Overall n (%)	CKD risk reported n (%)	No CKD risk reported n (%)
Total	203	106 (52.2)	97 (47.8)
Country of practice			
Canada	71 (35.0)	39 (36.8)	32 (33.0)
The United States	45 (22.2)	22 (20.8)	23 (23.7)
The United Kingdom	73 (36.0)	40 (37.7)	33 (34.0)
Other	11 (5.4)	3 (2.8)	8 (8.2)
Missing	3 (1.5)	2 (1.9)	1 (1.0)
Years in clinical practice			
< 20 years	110 (54.2)	55 (51.9)	55 (56.7)
≥ 20 years	93 (45.8)	51 (48.1)	42 (43.3)
AKI consultations per year			
< 40 consultations	45 (22.2)	23 (21.7)	22 (22.7)
≥ 40 consultations	158 (77.8)	83 (78.3)	75 (77.3)
Type of practice^a^			
Academic	158 (77.8)	85 (80.2)	73 (75.3)
Community/Non-Academic	44 (21.7)	21 (19.8)	23 (23.7)
Other	10 (4.9)	4 (3.8)	6 (6.2)

*Note.* AKI = acute kidney injury; CKD = chronic kidney disease.

a Note mutually exclusive; survey participants could select more than 1 type of clinical practice.

### Survey Responses Based on Patient Characteristics Presented in Clinical Vignettes

**Figure 2.**
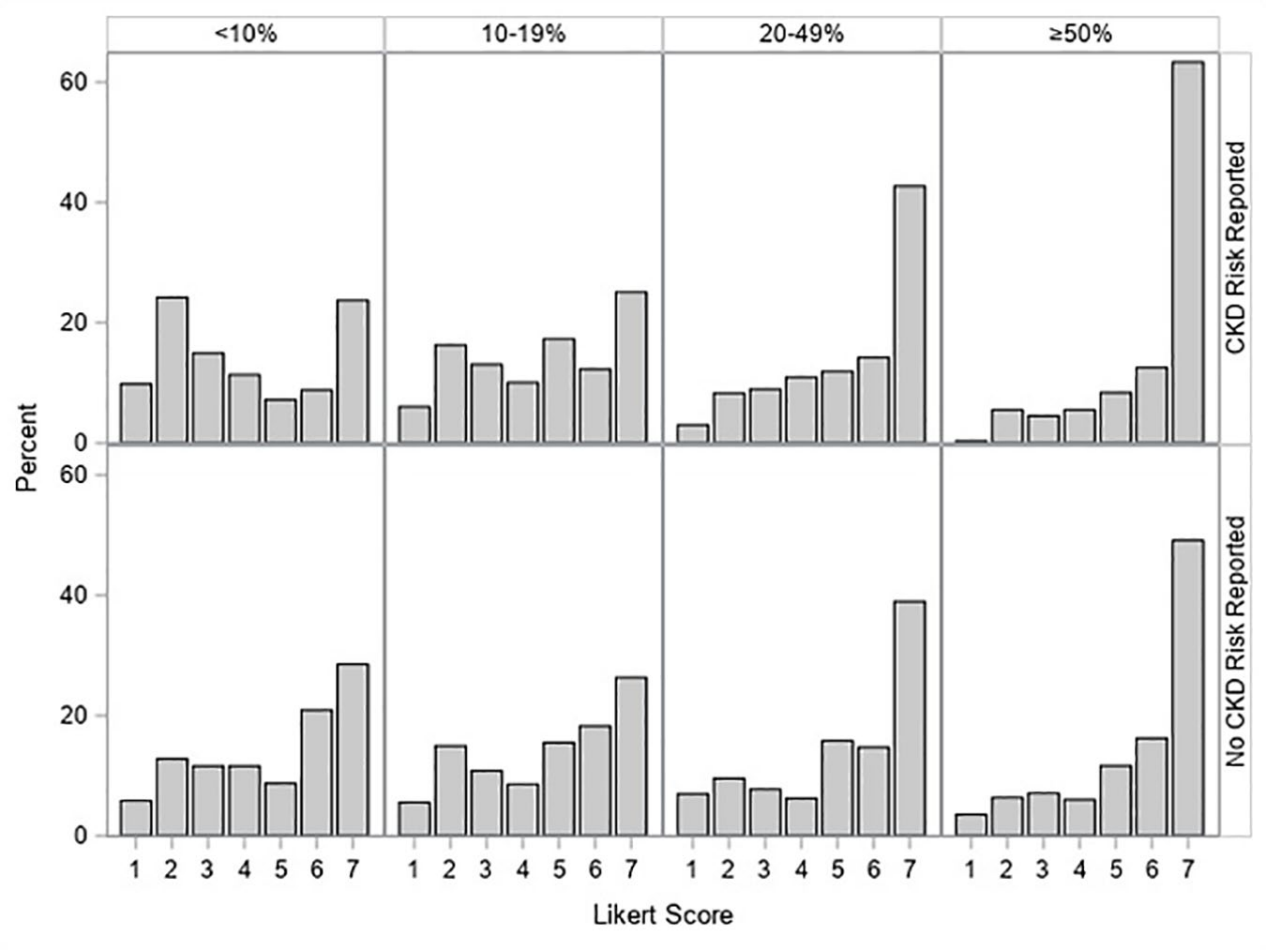
Distribution of Likert score responses by predicted risk of severe CKD and allocation to CKD risk reporting (top panel) or No CKD risk reporting (bottom panel). The predicted CKD risk categories are provided at the top of the figure. *Note.* The Likert scale ranged from 1 (definitely not) to 7 (definitely would) for the likelihood of recommending outpatient follow-up with a nephrology specialist. CKD = chronic kidney disease.

As the predicted risk of CKD presented in the clinical vignettes increased, the proportion of responses in higher Likert score categories also increased for both respondents who received CKD risk reporting and those who received no CKD risk reported (Figure 2). Among scenarios with 20% to 49% risk of CKD, almost 70% of Likert score responses were ≥5, while for scenarios with ≥ 50% risk of CKD, approximately 80% of responses were ≥5. The mean values and differences in Likert scores for participants who received CKD risk reporting versus those who received no CKD risk reporting are shown in Table 2. For scenarios with <10% predicted risk of CKD, respondents were significantly less likely to recommend outpatient nephrology specialist follow-up when the predicted risk of CKD was reported than when it was not reported (mean difference in Likert score = −0.71 [95% CI = −1.19, −0.23]). Conversely, among patient scenarios with ≥50% predicted risk of CKD, participants were significantly more likely to recommend outpatient nephrology specialist follow-up when the predicted risk was reported compared to when it was not reported (mean difference in Likert scale = 0.49 [95% CI = 0.04, 0.93]). Overall, there were no significant differences in responses based on respondents’ country, years in clinical practice, or the number of AKI consultations seen per year (S2 Supplementary Figure 1). Stratified analysis by country revealed a consistent trend in the effect across different regions (S3 Supplementary Table 1).

**Table 2.** Mean Differences in Likert Scores and Odds Ratios of Likert Score ≥ 5 for Recommending Nephrology Follow-up With Reporting vs No Reporting of the Predicted Risk of Severe CKD.

Predicted CKD risk category	CKD risk reported		No CKD risk reported		Mean difference (95% CI)	CKD risk reported—Likert score ≥ 5, n (%)	No CKD risk reported—Likert score ≥ 5, n (%)	Odds ratio (95% CI) for Likert score ≥ 5
	Number of responses	Mean score (95% CI)	Number of responses	Mean score (95% CI)				
<10%	194	4.01 (3.68, 4.34)	172	4.72 (4.37, 5.07)	−0.71 (−1.19, −0.23)	77 (39.7)	100 (58.1)	0.35 (0.16, 0.74)
10%-19%	399	4.51 (4.21, 4.81)	361	4.66 (4.34, 4.97)	−0.15 (−0.58, 0.29)	218 (54.6)	217 (60.1)	0.76 (0.39, 1.47)
20%-49%	302	5.32 (5.01, 5.63)	272	5.06 (4.74, 5.39)	0.26 (−0.19, 0.71)	208 (68.9)	189 (69.5)	1.10 (0.54, 2.24)
≥ 50%	311	6.06 (5.76, 6.37)	283	5.58 (5.26, 5.90)	0.49 (0.04, 0.93)	262 (84.2)	218 (77.0)	2.21 (1.02, 4.75)

*Note.* CKD = chronic kidney disease.

**Figure 3.**
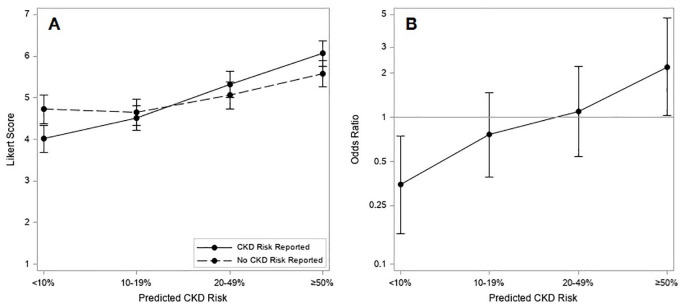
(A) Mean Likert score response by predicted risk of severe CKD and allocation to CKD risk reporting or no CKD risk reporting; (B) odds ratios for Likert score response ≥5 with CKD risk reporting vs no CKD risk reporting, by predicted CKD risk. *Note.* CKD = chronic kidney disease.

The proportion of responses with a Likert score ≥5 also increased for the vignettes with higher predicted risk of CKD (Figure 3A). The proportion of responses with a Likert score ≥5 and the corresponding odds ratios are reported in Table 2. At a predicted risk of CKD of <10%, the odds of a score ≥5 for recommending nephrologist specialist follow-up were lower when the CKD risk was reported than when not reported (OR = 0.35 [95% CI = 0.16, 0.74]), whereas with predicted risks of CKD of ≥50%, the odds of a score ≥5 for recommending nephrologist specialist follow-up were more than twice as high as when the risk of CKD was reported (OR = 2.21 [95% CI = 1.02, 4.75]) (Figure 3B).

### Individual Patient Characteristics and Likelihood of Recommending Nephrologist Follow-up

Patient characteristics and their relationship with mean Likert score for the likelihood of recommending nephrologist specialty follow-up are shown in S4 Supplementary Figure 2.

The highest mean Likert scores were obtained for the characteristics of baseline eGFR (mean = 6.1 [95% CI = 5.9, 6.2]), SCr at discharge (mean = 6.0 [95% CI = 5.9, 6.2]), albuminuria (mean = 6.0 [95% CI = 5.8, 6.2]), risk of advanced CKD (mean = 5.4 [95% CI = 5.2, 5.7]), multimorbidity (mean = 5.3 [95% CI = 5.1, 5.5]), heart failure (mean = 5.2 [95% CI = 5.0, 5.4]), patient age (mean = 5.2 [95% CI = 4.9, 5.4]), and AKI stage (mean = 5.1 [95% CI = 4.9, 5.4]).

## Discussion

In this survey study of nephrologists from the United States, the United Kingdom, and Canada, we found that the predicted risk of severe CKD influenced recommendations to provide nephrology specialist follow-up after a hospitalization with AKI. For patient scenarios with >20% predicted risk of severe CKD, participants indicated they would be likely to recommend nephrology specialist follow-up after hospitalization with AKI. Furthermore, when the predicted risk of severe CKD was below 10%, reporting the risk of CKD significantly reduced the likelihood of recommending outpatient nephrology specialist follow-up, whereas when the predicted risk of CKD was at or above 50%, reporting the predicted risk of CKD significantly increased the likelihood of recommending such follow-up. Nephrologists indicated that albuminuria, baseline eGFR, SCr at discharge, risk of advanced CKD, multimorbidity, heart failure, AKI stage, and patient age were the factors that most influenced their decisions about follow-up. These findings suggest that the use of validated risk models for CKD could be incorporated into clinical pathways to guide follow-up care after AKI, and that reporting the risk of severe CKD could serve as a decision support tool to improve the efficiency and appropriateness of care, by encouraging less use of nephrology resources for follow-up of low-risk patient, and greater use for high-risk patients. A growing body of literature demonstrates that nephrology specialists are rarely involved in the care of patients following AKI, yet that there may be opportunities to improve the quality of care and clinical outcomes following AKI.^15-19^ Several observational studies^20-22^ have reported that early nephrology follow-up care after AKI was associated with better patient outcomes including better kidney function,^23,24^ less progression of CKD,^21^ fewer kidney and cardiovascular events, and greater patient survival.^20,24,25^ Nephrology follow-up after AKI has additional potential benefits for cardiovascular disease management and risk reduction. Patients with AKI often have cardiovascular medications, such as angiotensin-converting enzyme inhibitors (ACEi) and SGLT2i, temporarily discontinued.^24,26^ Nephrology follow-up can facilitate timely reassessment and reintroduction of these medications, which may improve cardiovascular outcomes.^23,24^ A systematic review and meta-analysis found 22% lower mortality for patients with nephrology follow-up after AKI compared to those without.^25^ However, only observational studies were included and significant heterogeneity was observed among the included studies, limiting the strength of the evidence.^25^ Small randomized controlled trials (RCTs) conducted to date have been insufficient to demonstrate beneficial effects of nephrology follow-up on clinical outcomes.^10,27,28^ Larger, well-designed RCTs are needed to clarify the impact of nephrology follow-up on long-term outcomes.

Nephrologists, given their expertise in managing kidney and cardiovascular complications, are well-suited to enhance outpatient care for AKI survivors, providing appropriate monitoring for kidney disease progression, management of CKD complications, blood pressure control, medication reconciliation, and patient education on treatment options for kidney failure when needed.^21,23,29,30^ Despite these numerous potential benefits, outpatient nephrology specialist follow-up care remains relatively infrequent and there are too few nephrologists to care for all patients with AKI.^31-33^ In a large cohort study conducted in the US involving 156,733 adult patients with CKD, only 58% of those at high risk of progressing to kidney failure had a nephrology visit within 1 year of their risk being established.^31^ Similarly, a study from Alberta, Canada, involving 23 921 postdischarge patients with hospitalized AKI found that 21% of those with pre-existing CKD and 9% of those without CKD had nephrologist follow-up. In addition, only 52.7% with pre-existing CKD and 51.6% with de novo CKD were prescribed an RAAS (renin-angiotensin-aldosterone system) inhibitor suggesting opportunities for improved care.^34^

Our study found that in patient scenarios with a predicted risk of severe CKD greater than 20%, over half of participants rated these scenarios with a Likert scale 5 or higher, with a mean score also above 5 suggesting a heightened perception of need for nephrology care following AKI for patients at high risk of severe CKD. This response likely reflects an understanding of the serious implications of severe CKD and the need for careful management and intervention. Consequently, accurately predicting and communicating CKD risk may be clinically useful, as it can guide clinical decision-making and might help improve patient outcomes through timely and appropriate interventions. However, the translation of these findings into real clinical practice remains unclear, and there are conflicting views regarding whether specialist nephrologist care practices improve the quality of care provided by primary care physicians and other health care practitioners. Some studies have shown that nephrologists are more proficient in early recognition and management of severe CKD using evidence-based guidelines compared to general practitioners,^30,35^ while another study found that nurse practitioners provided better guideline-concordant care for CKD patients than primary care physicians alone or with nephrologists.^36^ Long-term interventional studies are needed to compare models of care for patients with AKI and high risk of CKD

Interestingly, our study found that in patient scenarios with a predicted risk of CKD risk less than 10%, nephrologists were significantly less likely to recommend follow-up when the CKD risk was reported, possibly because identification of a lower risk may reassure them, reducing the perceived necessity for specialist intervention. Conversely, in scenarios with a predicted risk of CKD ≥50%, nephrologists were more likely to recommend follow-up when the CKD risk was reported, underscoring a perception that nephrologist specialist care may be appropriate and valuable for such patients. These findings indicate that providing predicted CKD risk information can influence clinical decision-making, aligning recommendations more closely with patient risk. A previous Canadian survey study^37^ of nephrologists conducted in 2012 indicated they would definitely or probably recommend follow-up of patients with stage 3 AKI, particularly when accompanied by pre-existing CKD, heart failure, history of acute dialysis, and incomplete kidney function recovery. Furthermore, the study reported that the likelihood of nephrologists recommending follow-up was much higher than actual rates of nephrology follow-up in real-world practice. Our study expands on these findings by identifying albuminuria, baseline eGFR, SCr at discharge, risk of advanced CKD, multimorbidity, heart failure, AKI stage, and patient age as the most influential variables when deciding upon the need for nephrology follow-up. This understanding can inform clinical pathways for nephrology programs and the delivery of programs to prevent the progression of CKD and associated poor patient outcomes.^38-41^

## Strengths and Limitations

Strengths of this study include the participation of nephrologist from 3 countries, randomized allocation to versions of the survey with and without reporting of predicted CKD risk, and use of a risk prediction model that has been externally tested. However, there are limitations to the study. First, this survey was distributed only to nephrologists from high-income countries, which limits the generalizability of the findings to other regions, especially for countries with different health care systems. Differences in health care resources across countries may influence the applicability of the findings in other settings. Second, the survey relied on responses from nephrologists to hypothetical scenarios, which may differ from their practices in real-world clinical care where different incentives may operate. Clinical trials comparing processes and outcomes of care with, versus without, reporting predicted risk of CKD are needed to evaluate true clinical effects. Third, the study may be vulnerable to selection bias since participants who chose to respond to the survey may differ in significant ways from those who did not, potentially affecting the representativeness of responses obtained. Nephrologists who chose to respond to the survey may be more likely to have particular interests in AKI care. This could lead to more favorable responses to survey questions and an overestimation of the perceived benefits of nephrology follow-up from this study. Nonetheless, we obtained responses from nephrologists across a range of practice settings, volume, and years of practice, with consistent findings across these subgroups.

## Conclusions

This study demonstrates that nephrologists’ recommendations for outpatient follow-up after AKI significantly vary by the predicted risk of severe CKD, and that reporting the risk of CKD can affect their decision-making. The implementation of risk prediction models within clinical practice could be used to improve the alignment of nephrology care pathways with patient risk profiles.

## Supplemental Material

sj-docx-1-cjk-10.1177_20543581251336548 – Supplemental material for Nephrologist’s Perceptions of Risk of Severe Chronic Kidney Disease and Outpatient Follow-up After Hospitalization With AKI: Multinational Randomized Survey Study Supplemental material, sj-docx-1-cjk-10.1177_20543581251336548 for Nephrologist’s Perceptions of Risk of Severe Chronic Kidney Disease and Outpatient Follow-up After Hospitalization With AKI: Multinational Randomized Survey Study by Dilaram Acharya, Tayler D. Scory, Nusrat Shommu, Maoliosa Donald, Tyrone G. Harrison, Jonathan S. Murray, Simon Sawhney, Edward D. Siew, Neesh Pannu and Matthew T. James in Canadian Journal of Kidney Health and Disease
