# Supplementary material for: Nephrologist’s Perceptions of Risk of Severe Chronic Kidney Disease and Outpatient Follow-up After Hospitalization With AKI: Multinational Randomized Survey Study
Source: Can J Kidney Health Dis. 2025 Apr 30;12:20543581251336548. doi: 10.1177/20543581251336548 (PMC12046161; doi:10.1177/20543581251336548)
Supplement: sj-docx-1-cjk-10.1177_20543581251336548 – Supplemental material for Nephrologist’s Perceptions of Risk of Severe Chronic Kidney Disease and Outpatient Follow-up After Hospitalization With AKI: Multinational Randomized Survey Study [file sj-docx-1-cjk-10.1177_20543581251336548.docx]

**SUPPLIMENTARY INFORMATION**

**S1 Survey questionnaire with CKD risk reported** (italicized sections indicate the risk information, which was only included in the version of the survey distributed to nephrologists who were randomly allocated to receive the CKD risk reported)

Nephrology Follow-up after Hospitalization with Acute Kidney Injury: An International Survey of Nephrologists

Dear Participant,

Thank you for your participation in the following survey. Your participation is voluntary, and you are under no obligation to complete the survey. The purpose of this survey is to better understand the factors that influence nephrologist’s decisions to recommend nephrology specialist follow-up to patients after hospitalization with acute kidney injury. Your responses will be completely confidential and anonymous. Responses will be analyzed in aggregate and at no time will individual responses be made available to anyone. This survey has been approved by the Conjoint Health Research Ethics Board at the University of Calgary. Compiled results of this survey will be presented in peer-reviewed publications and scientific presentations.

 By checking the box below, and completing the survey, you are consenting to the use of your answers in academic research. There are five demographic questions followed by three scenarios, each comprised of four questions. 

 The survey should take no more than 10 minutes to complete. Thank you for participating. If you have any questions, please do not hesitate to contact:

 Dr. Matthew James MD, PhD, FRCPC
 Division of Nephrology, University of Calgary
 Phone: 403-220-2465
 Fax: 403-210-6660-
 Email: mjames@ucalgary.ca

**I understand the above stated purpose of this survey and consent to the use of my responses for the purpose of academic research:**

- Yes
- No

**Please Enter the Country o your practice**

________________________________________________________________

**Please Enter the Province, County, or State of your practice**

________________________________________________________________

**How many years have you been in clinical nephrology practice?**

- <5 (1)
- 5-9 (2)
- 10-19 (3)
- 20-29 (4)
- 30-39 (5)
- >40 (6)

**Approximately, how many consultations for acute kidney injury do you see each year?**

- <20 (1)
- 20-39 (2)
- 40-59 (3)
- >60 (4)

I**s your clinical practice:**
(please check all that apply)

- Academic (I work in a teaching hospital with residents and fellows)
- Private
- Other (3) __________________________________________________

Arm 1: With CKD Risk Score

**Scenario A**
 You are seeing a patient in hospital on the day of planned discharge home. You have been following this 55 year old woman during her admission with acute kidney injury secondary to septic shock. Her comorbidities include hypertension and dyslipidemia. Her urine albumin to creatinine (ACR) before this hospitalization was 32 mg/mmol.

**A1.** Assuming her baseline serum creatinine was 88 umol/L [1.0 mg/dl], corresponding eGFR 67 mL/min/1.73m2 prior to hospitalization, peak serum creatinine in hospital was 246 umol/L [2.8 mg/dl], corresponding to KDIGO stage 2 AKI, and her serum creatinine today is 114 umol/L [1.3 mg/dl] on the day of discharge*, which carries to a predicted risk of having category G4* (eGFR<30 mL/min/1.73m2) or greater CKD of 10% in the next year based on these 6 variables*.

 [** Category G4 CKD Risk Score Calculation and reference*](https://qxmd.com/calculate/calculator_451/advanced-ckd-after-aki-risk-index)

|  | Definitely Not (1) | 2 | 3 | 4 | 5 | 6 | Definitely Would (7) |
| --- | --- | --- | --- | --- | --- | --- | --- |
| How likely are you to recommend outpatient follow-up with a nephrology specialist? |  |  |  |  |  |  |  |

**A2.** Assuming her baseline serum creatinine was 106 umol/L [1.2 mg/dl], corresponding eGFR 53 mL/min/1.73m2 prior to hospitalization, peak serum creatinine in hospital was 297 umol/L [3.4 mg/dl], corresponding to KDIGO stage 2 AKI, and her serum creatinine today is 114 umol/L [1.3 mg/dl] on the day of discharge, *which carries to a predicted risk of having category G4 (eGFR<30 mL/min/1.73m2) or greater CKD of 20% in the next year based on these 6 variables.*

|  | Definitely Not (1) | 2 | 3 | 4 | 5 | 6 | Definitely Would (7) |
| --- | --- | --- | --- | --- | --- | --- | --- |
| How likely are you to recommend outpatient follow-up with a nephrology specialist? |  |  |  |  |  |  |  |

**A3.** Assuming her baseline serum creatinine was 132 umol/L [1.5 mg/dl], corresponding eGFR 41 mL/min/1.73m2 prior to hospitalization, peak serum creatinine in hospital was 528 umol/L [6.0 mg/dl], corresponding to KDIGO stage 3 AKI, and her serum creatinine today is 167 umol/L [1.9 mg/dl] on the day of discharge, *which carries to a predicted risk of having category G4( (eGFR<30 mL/min/1.73m2) or greater CKD of 70% in the next year based on these 6 variables.*

|  | Definitely Not (1) | 2 | 3 | 4 | 5 | 6 | Definitely Would (7) |
| --- | --- | --- | --- | --- | --- | --- | --- |
| How likely are you to recommend outpatient follow-up with a nephrology specialist? |  |  |  |  |  |  |  |

**A4.** Assuming her baseline serum creatinine was 150 umol/L [1.7 mg/dl], corresponding eGFR 35 mL/min/1.73m2 prior to hospitalization, peak serum creatinine in hospital was 602 umol/L [6.8 mg/dl], corresponding to KDIGO stage 3 AKI, and her serum creatinine today is 150 umol/L [1.7 mg/dl] on the day of discharge, *which carries to a predicted risk of having category G4 (eGFR<30 mL/min/1.73m2) or greater CKD of 50% in the next year based on these 6 variables*

|  | Definitely Not (1) | 2 | 3 | 4 | 5 | 6 | Definitely Would (7) |
| --- | --- | --- | --- | --- | --- | --- | --- |
| How likely are you to recommend outpatient follow-up with a nephrology specialist? |  |  |  |  |  |  |  |

**Scenario B**
You are seeing a patient in hospital on the day of planned discharge home. You have been following this 75-year-old man during his admission with acute kidney injury secondary to septic shock. His comorbidities include hypertension and dyslipidemia. His urine albumin to creatinine (ACR) before this hospitalization was 58 mg/mmol.

**B1.** Assuming his baseline serum creatinine was 107 umol/L [1.2 mg/dl], corresponding eGFR 62 mL/min/1.73m2 prior to hospitalization, peak serum creatinine in hospital was 412 umol/L [4.7 mg/dl], corresponding to KDIGO stage 3 AKI, and his serum creatinine today is 115 umol/L [1.3 mg/dl] on the day of discharge, *which carries to a predicted risk of having category G4 (eGFR<30 mL/min/1.73m2) or greater CKD of 12% in the next year based on these 6 variables.*

|  | Definitely Not (1) | 2 | 3 | 4 | 5 | 6 | Definitely Would (7) |
| --- | --- | --- | --- | --- | --- | --- | --- |
| How likely are you to recommend outpatient follow-up with a nephrology specialist? |  |  |  |  |  |  |  |

**B2.** Assuming his baseline serum creatinine was 107 umol/L [1.2 mg/dl], corresponding eGFR 62 mL/min/1.73m2 prior to hospitalization, peak serum creatinine in hospital was 349 umol/L [3.9 mg/dl], corresponding to KDIGO stage 3 AKI, and his serum creatinine today is 142 umol/L [1.6 mg/dl] on the day of discharge, *which carries to a predicted risk of having category G4 (eGFR<30 mL/min/1.73m2) or greater CKD of 15% in the next year based on these 6 variables.*

|  | Definitely Not (1) | 2 | 3 | 4 | 5 | 6 | Definitely Would (7) |
| --- | --- | --- | --- | --- | --- | --- | --- |
| How likely are you to recommend outpatient follow-up with a nephrology specialist? (1) |  |  |  |  |  |  |  |

**B3.** Assuming his baseline serum creatinine was 158 umol/L [1.8 mg/dl], corresponding eGFR 39 mL/min/1.73m2 prior to hospitalization, peak serum creatinine in hospital was 862 umol/L [9.8 mg/dl], corresponding to KDIGO stage 3 AKI, and his serum creatinine today is 156 umol/L [1.8 mg/dl] on the day of discharge, *which carries to a predicted risk of having category G4 (eGFR<30 mL/min/1.73m2) or greater CKD of 35% in the next year based on these 6 variables.*

|  | Definitely Not (1) | 2 | 3 | 4 | 5 | 6 | Definitely Would (7) |
| --- | --- | --- | --- | --- | --- | --- | --- |
| How likely are you to recommend outpatient follow-up with a nephrology specialist? |  |  |  |  |  |  |  |

**B4.** Assuming his baseline serum creatinine was 162 umol/L [1.8 mg/dl], corresponding eGFR 38 mL/min/1.73m2 prior to hospitalization, peak serum creatinine in hospital was 502 umol/L [5.7 mg/dl], corresponding to KDIGO stage 3 AKI, and his serum creatinine today is 167 umol/L [1.9 mg/dl] on the day of discharge, *which carries to a predicted risk of having category G4 (eGFR<30 mL/min/1.73m2) or greater CKD of 65% in the next year based on these 6 variables.*

|  | Definitely Not (1) | 2 | 3 | 4 | 5 | 6 | Definitely Would (7) |
| --- | --- | --- | --- | --- | --- | --- | --- |
| How likely are you to recommend outpatient follow-up with a nephrology specialist? |  |  |  |  |  |  |  |

**Scenario C**
You are seeing a patient in hospital on the day of planned discharge home. You have been following this 36-year-old woman during her admission with acute kidney injury secondary to septic shock. Her comorbidities include hypertension and dyslipidemia. Her urine albumin to creatinine (ACR) before this hospitalization was never measured.

**C1.** Assuming her baseline serum creatinine was 97 umol/L [1.1 mg/dl], corresponding eGFR 56 mL/min/1.73m2 prior to hospitalization, peak serum creatinine in hospital was 1010 umol/L [11.0 mg/dl], corresponding to KDIGO stage 3 AKI, and her serum creatinine today is 102 umol/L [1.1 mg/dl] on the day of discharge, *which carries to a predicted risk of having category G4 (eGFR<30 mL/min/1.73m2) or greater CKD of 4% in the next year based on these 6 variables.*

|  | Definitely Not (1) | 2 | 3 | 4 | 5 | 6 | Definitely Would (7) |
| --- | --- | --- | --- | --- | --- | --- | --- |
| How likely are you to recommend outpatient follow-up with a nephrology specialist? |  |  |  |  |  |  |  |

**C2.** Assuming her baseline serum creatinine was 97 umol/L [1.1 mg/dl], corresponding eGFR 56 mL/min/1.73m2 prior to hospitalization, peak serum creatinine in hospital was 485 umol/L [5.5 mg/dl], corresponding to KDIGO stage 3 AKI, and her serum creatinine today is 115 umol/L [1.3 mg/dl] on the day of discharge, *which carries to a predicted risk of having category G4 (eGFR<30 mL/min/1.73m2) or greater CKD of 9% in the next year based on these 6 variables.*

|  | Definitely Not (1) | 2 | 3 | 4 | 5 | 6 | Definitely Would (7) |
| --- | --- | --- | --- | --- | --- | --- | --- |
| How likely are you to recommend outpatient follow-up with a nephrology specialist? |  |  |  |  |  |  |  |

**C3.** Assuming her baseline serum creatinine was 132 umol/L [1.5 mg/dl], corresponding eGFR 39 mL/min/1.73m2 prior to hospitalization, peak serum creatinine in hospital was 402 umol/L [4.6 mg/dl], corresponding to KDIGO stage 3 AKI, and her serum creatinine today is 129 umol/L [1.5 mg/dl] on the day of discharge, *which carries to a predicted risk of having category G4 (eGFR<30 mL/min/1.73m2) or greater CKD of 15% in the next year based on these 6 variables.*

|  | Definitely Not (1) | 2 | 3 | 4 | 5 | 6 | Definitely Would (7) |
| --- | --- | --- | --- | --- | --- | --- | --- |
| How likely are you to recommend outpatient follow-up with a nephrology specialist? |  |  |  |  |  |  |  |

**C4.** Assuming her baseline serum creatinine was 160 umol/L [1.8 mg/dl], corresponding eGFR 31 mL/min/1.73m2 prior to hospitalization, peak serum creatinine in hospital was 475 umol/L [5.4 mg/dl], corresponding to KDIGO stage 3 AKI, and her serum creatinine today is 164 umol/L [1.9 mg/dl] on the day of discharge, *which carries to a predicted risk of having category G4 (eGFR<30 mL/min/1.73m2) or greater CKD of 25% in the next year based on these 6 variables.*

|  | Definitely Not (1) | 2 | 3 | 4 | 5 | 6 | Definitely Would (7) |
| --- | --- | --- | --- | --- | --- | --- | --- |
| How likely are you to recommend outpatient follow-up with a nephrology specialist? |  |  |  |  |  |  |  |

Arm 2: Without CKD Risk Score

**Scenario A**
You are seeing a patient in hospital on the day of planned discharge home. You have been following this 55 year old woman during her admission with acute kidney injury secondary to septic shock. Her comorbidities include hypertension and dyslipidemia. Her urine albumin to creatinine (ACR) before this hospitalization was 32 mg/mmol.

**A1.** Assuming her baseline serum creatinine was 88 umol/L [1.0 mg/dl], corresponding eGFR 67 mL/min/1.73m2 prior to hospitalization, peak serum creatinine in hospital was 246 umol/L [2.8 mg/dl], corresponding to KDIGO stage 2 AKI, and her serum creatinine today is 114 umol/L [1.3 mg/dl] on the day of discharge.

|  | Definitely Not (1) | 2 | 3 | 4 | 5 | 6 | Definitely Would (7) |
| --- | --- | --- | --- | --- | --- | --- | --- |
| How likely are you to recommend outpatient follow-up with a nephrology specialist? |  |  |  |  |  |  |  |

**A2.** Assuming her baseline serum creatinine was 106 umol/L [1.2 mg/dl], corresponding eGFR 53 mL/min/1.73m2 prior to hospitalization, peak serum creatinine in hospital was 297 umol/L [3.4 mg/dl], corresponding to KDIGO stage 2 AKI, and her serum creatinine today is 114 umol/L [1.3 mg/dl] on the day of discharge.

|  | Definitely Not (1) | 2 | 3 | 4 | 5 | 6 | Definitely Would (7) |
| --- | --- | --- | --- | --- | --- | --- | --- |
| How likely are you to recommend outpatient follow-up with a nephrology specialist? |  |  |  |  |  |  |  |

**A3.** Assuming her baseline serum creatinine was 132 umol/L [1.5 mg/dl], corresponding eGFR 41 mL/min/1.73m2 prior to hospitalization, peak serum creatinine in hospital was 528 umol/L [6.0 mg/dl], corresponding to KDIGO stage 3 AKI, and her serum creatinine today is 167 umol/L [1.9 mg/dl] on the day of discharge.

|  | Definitely Not (1) | 2 | 3 | 4 | 5 | 6 | Definitely Would (7) |
| --- | --- | --- | --- | --- | --- | --- | --- |
| How likely are you to recommend outpatient follow-up with a nephrology specialist? |  |  |  |  |  |  |  |

**A4.** Assuming her baseline serum creatinine was 150 umol/L [1.7 mg/dl], corresponding eGFR 35 mL/min/1.73m2 prior to hospitalization, peak serum creatinine in hospital was 602 umol/L [6.8 mg/dl], corresponding to KDIGO stage 3 AKI, and her serum creatinine today is 150 umol/L [1.7 mg/dl] on the day of discharge.

|  | Definitely Not (1) | 2 | 3 | 4 | 5 | 6 | Definitely Would (7) |
| --- | --- | --- | --- | --- | --- | --- | --- |
| How likely are you to recommend outpatient follow-up with a nephrology specialist? |  |  |  |  |  |  |  |

**Scenario B**
You are seeing a patient in hospital on the day of planned discharge home. You have been following this 75 year old man during his admission with acute kidney injury secondary to septic shock. His comorbidities include hypertension and dyslipidemia. His urine albumin to creatinine (ACR) before this hospitalization was 58 mg/mmol.

**B1.** Assuming his baseline serum creatinine was 107 umol/L [1.2 mg/dl], corresponding eGFR 62 mL/min/1.73m2 prior to hospitalization, peak serum creatinine in hospital was 412 umol/L [4.7 mg/dl], corresponding to KDIGO stage 3 AKI, and his serum creatinine today is 115 umol/L [1.3 mg/dl] on the day of discharge.

|  | Definitely Not (1) | 2 | 3 | 4 | 5 | 6 | Definitely Would (7) |
| --- | --- | --- | --- | --- | --- | --- | --- |
| How likely are you to recommend outpatient follow-up with a nephrology specialist? |  |  |  |  |  |  |  |

**B2.** Assuming his baseline serum creatinine was 107 umol/L [1.2 mg/dl], corresponding eGFR 62 mL/min/1.73m2 prior to hospitalization, peak serum creatinine in hospital was 349 umol/L [3.9 mg/dl], corresponding to KDIGO stage 3 AKI, and his serum creatinine today is 142 umol/L [1.6 mg/dl] on the day of discharge.

|  | Definitely Not (1) | 2 | 3 | 4 | 5 | 6 | Definitely Would (7) |
| --- | --- | --- | --- | --- | --- | --- | --- |
| How likely are you to recommend outpatient follow-up with a nephrology specialist? |  |  |  |  |  |  |  |

**B3.** Assuming his baseline serum creatinine was 158 umol/L [1.8 mg/dl], corresponding eGFR 39 mL/min/1.73m2 prior to hospitalization, peak serum creatinine in hospital was 862 umol/L [9.8 mg/dl], corresponding to KDIGO stage 3 AKI, and his serum creatinine today is 156 umol/L [1.8 mg/dl] on the day of discharge.

|  | Definitely Not (1) | 2 | 3 | 4 | 5 | 6 | Definitely Would (7) |
| --- | --- | --- | --- | --- | --- | --- | --- |
| How likely are you to recommend outpatient follow-up with a nephrology specialist? |  |  |  |  |  |  |  |

**B4.** Assuming his baseline serum creatinine was 162 umol/L [1.8 mg/dl], corresponding eGFR 38 mL/min/1.73m2 prior to hospitalization, peak serum creatinine in hospital was 502 umol/L [5.7 mg/dl], corresponding to KDIGO stage 3 AKI, and his serum creatinine today is 167 umol/L [1.9 mg/dl] on the day of discharge.

|  | Definitely Not (1) | 2 | 3 | 4 | 5 | 6 | Definitely Would (7) |
| --- | --- | --- | --- | --- | --- | --- | --- |
| How likely are you to recommend outpatient follow-up with a nephrology specialist? |  |  |  |  |  |  |  |

**Scenario C**
You are seeing a patient in hospital on the day of planned discharge home. You have been following this 36 year old woman during her admission with acute kidney injury secondary to septic shock. Her comorbidities include hypertension and dyslipidemia. Her urine albumin to creatinine (ACR) before this hospitalization was never measured.

**C1.** Assuming her baseline serum creatinine was 97 umol/L [1.1 mg/dl], corresponding eGFR 56 mL/min/1.73m2 prior to hospitalization, peak serum creatinine in hospital was 1010 umol/L [11.0 mg/dl], corresponding to KDIGO stage 3 AKI, and her serum creatinine today is 102 umol/L [1.1 mg/dl] on the day of discharge.

|  | Definitely Not (1) | 2 | 3 | 4 | 5 | 6 | Definitely Would (7) |
| --- | --- | --- | --- | --- | --- | --- | --- |
| How likely are you to recommend outpatient follow-up with a nephrology specialist? |  |  |  |  |  |  |  |

**C2.** Assuming her baseline serum creatinine was 97 umol/L [1.1 mg/dl], corresponding eGFR 56 mL/min/1.73m2 prior to hospitalization, peak serum creatinine in hospital was 485 umol/L [5.5 mg/dl], corresponding to KDIGO stage 3 AKI, and her serum creatinine today is 115 umol/L [1.3 mg/dl] on the day of discharge.

|  | Definitely Not (1) | 2 | 3 | 4 | 5 | 6 | Definitely Would (7) |
| --- | --- | --- | --- | --- | --- | --- | --- |
| How likely are you to recommend outpatient follow-up with a nephrology specialist? |  |  |  |  |  |  |  |

**C3.** Assuming her baseline serum creatinine was 132 umol/L [1.5 mg/dl], corresponding eGFR 39 mL/min/1.73m2 prior to hospitalization, peak serum creatinine in hospital was 402 umol/L [4.6 mg/dl], corresponding to KDIGO stage 3 AKI, and her serum creatinine today is 129 umol/L [1.5 mg/dl] on the day of discharge.

|  | Definitely Not (1) | 2 | 3 | 4 | 5 | 6 | Definitely Would (7) |
| --- | --- | --- | --- | --- | --- | --- | --- |
| How likely are you to recommend outpatient follow-up with a nephrology specialist? |  |  |  |  |  |  |  |

**C4.** Assuming her baseline serum creatinine was 160 umol/L [1.8 mg/dl], corresponding eGFR 31 mL/min/1.73m2 prior to hospitalization, peak serum creatinine in hospital was 475 umol/L [5.4 mg/dl], corresponding to KDIGO stage 3 AKI, and her serum creatinine today is 164 umol/L [1.9 mg/dl] on the day of discharge.

|  | Definitely Not (1) | 2 | 3 | 4 | 5 | 6 | Definitely Would (7) |
| --- | --- | --- | --- | --- | --- | --- | --- |
| How likely are you to recommend outpatient follow-up with a nephrology specialist? |  |  |  |  |  |  |  |

**Please indicate the degree to which each of the following factors affects your decision to arrange outpatient follow-up of a patient after a hospitalization with AKI:**

|  | Not At All (1) | 2 | 3 | 4 | 5 | 6 | Very Much (7) |
| --- | --- | --- | --- | --- | --- | --- | --- |
| Age of patient |  |  |  |  |  |  |  |
| Sex of patient |  |  |  |  |  |  |  |
| Presence of albuminuria |  |  |  |  |  |  |  |
| Baseline eGFR |  |  |  |  |  |  |  |
| Severity of AKI (AKI stage) |  |  |  |  |  |  |  |
| Serum creatinine at time of discharge |  |  |  |  |  |  |  |
| Predicted risk of advanced CKD (Category G4 or greater) |  |  |  |  |  |  |  |
| Presence of comorbidity |  |  |  |  |  |  |  |
| Distance between home and nephrology Centre |  |  |  |  |  |  |  |
| Socioeconomic status of patient |  |  |  |  |  |  |  |
| Patient has a primary care physician for follow-up |  |  |  |  |  |  |  |
| Patient has a specialty physician for follow-up |  |  |  |  |  |  |  |
| Patient not prescribed guideline-recommended medication(s) at discharge |  |  |  |  |  |  |  |
| Other |  |  |  |  |  |  |  |


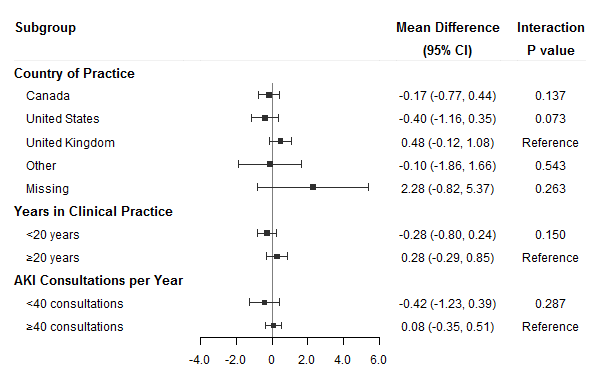


**S2 Supplementary Figure 1**: Effect of reporting predicted CKD risk on mean Likert score, by subgroups (n = 203)

**S3 Supplementary Table 1:** Mean Likert score response at each predicted CKD risk category, by allocation to CKD risk reporting versus no CKD risk reporting and country of practice

| Category | CKD Risk Reported Mean (95% CI) | No CKD Risk Reported Mean (95% CI) | Mean Difference  (95% CI) |
| --- | --- | --- | --- |
| Canada | | | |
| Predicted CKD Risk |  |  |  |
| < 10% | 3.67 (3.17, 4.16) | 4.81 (4.26, 5.35) | -1.14 (-1.87, -0.40) |
| 10 – 19% | 4.51 (4.06, 4.96) | 4.64 (4.15, 5.13) | -0.13 (-0.79, 0.54) |
| 20 – 49% | 5.41 (4.95, 5.88) | 5.36 (4.85, 5.87) | 0.05 (-0.63, 0.74) |
| ≥ 50% | 6.28 (5.82, 6.74) | 5.85 (5.34, 6.36) | 0.43 (-0.25, 1.11) |
| United States | | | |
| Predicted CKD Risk |  |  |  |
| < 10% | 4.27 (3.68, 4.87) | 5.25 (4.65, 5.85) | -0.98 (-1.83, -0.13) |
| 10 – 19% | 5.08 (4.53, 5.63) | 5.48 (4.93, 6.02) | -0.40 (-1.17, 0.38) |
| 20 – 49% | 5.77 (5.21, 6.34) | 5.78 (5.22, 6.33) | -0.00 (-0.80, 0.79) |
| ≥ 50% | 6.39 (5.83, 6.96) | 6.59 (6.04, 7.15) | -0.20 (-0.99, 0.60) |
| United Kingdom | | | |
| Predicted CKD Risk |  |  |  |
| < 10% | 4.32 (3.76, 4.88) | 4.42 (3.80, 5.05) | -0.10 (-0.95, 0.74) |
| 10 – 19% | 4.20 (3.70, 4.71) | 3.99 (3.43, 4.55) | 0.21 (-0.54, 0.96) |
| 20 – 49% | 4.97 (4.45, 5.49) | 4.24 (3.66, 4.82) | 0.73 (-0.04, 1.51) |
| ≥ 50% | 5.64 (5.13, 6.16) | 4.53 (3.96, 5.10) | 1.12 (0.35, 1.88) |
| Other/Missing | | | |
| Predicted CKD Risk |  |  |  |
| < 10% | 3.28 (1.42, 5.14) | 4.11 (2.81, 5.41) | -0.84 (-3.11, 1.44) |
| 10 – 19% | 4.55 (2.92, 6.18) | 5.08 (3.87, 6.28) | -0.52 (-2.55, 1.50) |
| 20 – 49% | 5.52 (3.88, 7.16) | 5.15 (3.93, 6.36) | 0.37 (-1.67, 2.42) |
| ≥ 50% | 6.27 (4.65, 7.90) | 5.83 (4.62, 7.04) | 0.44 (-1.58, 2.47) |


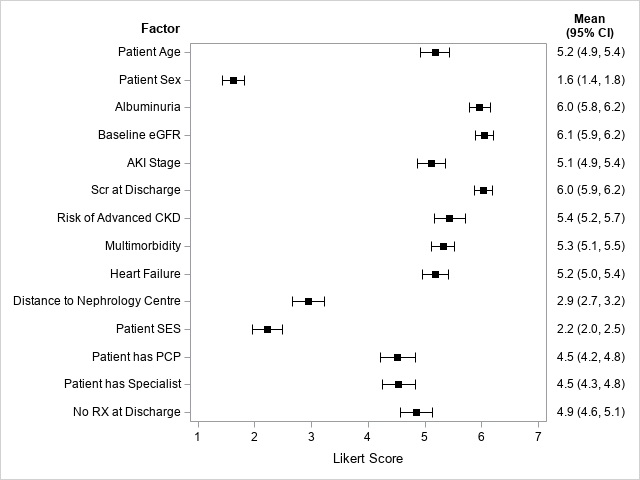


**S4 Supplementary Figure 2:** Mean Likert Score Responses of the Degree to Which Factors of Interest Affect Nephrologists’ Decisions to Arrange Outpatient Nephrology Follow-up Care after AKI (n = 203).

Abbreviations: CKD, chronic kidney disease; PCP, primary care physician; RX, prescription; SES, socioeconomic status.
